# Roles, Users, Benefits, and Limitations of Chatbots in Health Care: Rapid Review

**DOI:** 10.2196/56930

**Published:** 2024-07-23

**Authors:** Moustafa Laymouna, Yuanchao Ma, David Lessard, Tibor Schuster, Kim Engler, Bertrand Lebouché

**Affiliations:** 1 Department of Family Medicine Faculty of Medicine and Health Sciences McGill University Montreal, QC Canada; 2 Centre for Outcomes Research and Evaluation Research Institute of the McGill University Health Centre Montreal, QC Canada; 3 Infectious Diseases and Immunity in Global Health Program Research Institute of McGill University Health Centre Montreal, QC Canada; 4 Chronic and Viral Illness Service Division of Infectious Disease, Department of Medicine McGill University Health Centre Montreal, QC Canada; 5 Department of Biomedical Engineering Polytechnique Montréal Montreal, QC Canada

**Keywords:** chatbot, conversational agent, conversational assistant, user-computer interface, digital health, mobile health, electronic health, telehealth, artificial intelligence, AI, health information technology

## Abstract

**Background:**

Chatbots, or *conversational agents*, have emerged as significant tools in health care, driven by advancements in artificial intelligence and digital technology. These programs are designed to simulate human conversations, addressing various health care needs. However, no comprehensive synthesis of health care chatbots’ roles, users, benefits, and limitations is available to inform future research and application in the field.

**Objective:**

This review aims to describe health care chatbots’ characteristics, focusing on their diverse roles in the health care pathway, user groups, benefits, and limitations.

**Methods:**

A rapid review of published literature from 2017 to 2023 was performed with a search strategy developed in collaboration with a health sciences librarian and implemented in the MEDLINE and Embase databases. Primary research studies reporting on chatbot roles or benefits in health care were included. Two reviewers dual-screened the search results. Extracted data on chatbot roles, users, benefits, and limitations were subjected to content analysis.

**Results:**

The review categorized chatbot roles into 2 themes: *delivery of remote health services*, including patient support, care management, education, skills building, and health behavior promotion, and *provision of administrative assistance to health care providers*. User groups spanned across patients with chronic conditions as well as patients with cancer; individuals focused on lifestyle improvements; and various demographic groups such as women, families, and older adults. Professionals and students in health care also emerged as significant users, alongside groups seeking mental health support, behavioral change, and educational enhancement. The benefits of health care chatbots were also classified into 2 themes: *improvement of health care quality* and *efficiency and cost-effectiveness in health care delivery*. The identified limitations encompassed ethical challenges, medicolegal and safety concerns, technical difficulties, user experience issues, and societal and economic impacts.

**Conclusions:**

Health care chatbots offer a wide spectrum of applications, potentially impacting various aspects of health care. While they are promising tools for improving health care efficiency and quality, their integration into the health care system must be approached with consideration of their limitations to ensure optimal, safe, and equitable use.

## Introduction

### Background

In the dynamic landscape of IT and digital communication, chatbots—known as *conversational agents*—stand at the forefront, revolutionizing interactions between technology and human users. Chatbots are computer programs designed to simulate conversation through text, image, audio, or video messaging with human users on platforms such as websites, smartphone apps, or stand-alone computer software [1-47]. Originating from the concept *ChatterBot*, coined in 1994 [48], chatbots have undergone substantial evolution in their functionality and application.

The evolution of chatbots represents a significant technological leap, transitioning from reliance on predefined, rule-based scripted conversations to the sophisticated use of natural language processing and artificial intelligence (AI). By leveraging natural language processing and AI, chatbots have become capable of understanding and appropriately responding to user requests [49,50]. Their versatility has facilitated applications in a variety of sectors such as education, e-commerce, finance, news, health care, and entertainment. Popular instances of these applications include Amazon’s Alexa [51], Apple’s Siri [52], Google Assistant [53], Microsoft’s Cortana [54], and Samsung’s Bixby [55].

A notable advancement in the field of chatbots has been the integration of generative AI and large language models (LLMs) such as ChatGPT [56-58]. They have the capability to generate human-like text, enabling more natural and informative interactions [56-58]. However, their application in health care is still emerging. The risk of misinformation and errors is a significant concern [59,60], particularly in health care where accuracy is critical. The *one-size-fits-all* approach of LLMs may not align well with the nuanced needs of patient-centered care in the health sector [59].

The promise of chatbots in health care is considerable, offering potential for more efficient, cost-effective, and high-quality care [61-65], as well as their broad spectrum of uses and acceptability [66,67]. The use of chatbots to access and deliver health care services seems to be on the rise [23,68-70], granting them multiple potential roles in prevention, diagnosis, and support with care and treatment, with possible impacts on the whole health care system.

Despite the potential benefits, health care chatbots face unique challenges [71-74]. The need for highly specialized and context-sensitive advice is paramount. Generic responses from current chatbot models often overlook individual health profiles and local health contexts, which are crucial for patient care [75].

While a wide range of health care chatbot reviews have been conducted—demonstrating the versatility of chatbots in areas such as genetic cancer risk assessment [44]; oncological care [9,11,24,25]; sexual and reproductive health [35,45]; preconception, pregnancy, and postpartum health [36]; support for smoking cessation [38]; management of weight [39] and chronic conditions [6,9,20,40]; vaccine communication [26]; and broader health care acceptability [27]—these reviews often exhibit significant limitations in scope and depth. They tend to concentrate narrowly on specific applications such as rehabilitation for neurological conditions [28], mental health support [4,8,12-17,29,30,41,42], health behavior change [31-33,37], the language used in health communication by chatbots [43], and the use of chatbots in the COVID-19 public health response [44], leading to a fragmented understanding of chatbots’ roles in health care; for instance, while some reviews [3,7] offer insights, they do not encompass a comprehensive evaluation of the broader implications of chatbots, particularly in diverse contexts. By contrast, other reviews [5,30] concentrate extensively on technical aspects and AI algorithms [24,25,75,76]; yet, this focus tends to overshadow a detailed exploration of the impact these technologies have on health care outcomes.

### Objectives

This approach has left significant gaps in the literature. There is an evident need for an integrative overview that thoroughly analyzes the varied roles of chatbots across different health care applications, capturing new trends and advancements. Furthermore, the interactions and benefits of health care chatbots for diverse demographic groups, especially those who are underrepresented, are underexplored. There is also a conspicuous absence of a deeper understanding of the potential benefits and practical limitations of health care chatbots in various contexts.

Therefore, the objectives of this review are to bridge these existing knowledge gaps. Our review aims to provide a comprehensive exploration of chatbots’ functional roles, analyze the specific populations they serve, and examine in detail their potential and reported benefits, as well as the limitations of these innovative tools in health care. This endeavor will offer a more holistic and nuanced understanding of chatbots in the health care sector, addressing critical areas overlooked in previous studies.

## Methods

### Design and Search Strategy

This study is a rapid review, which refers to an accelerated, resource-efficient process of knowledge synthesis through streamlining or omitting specific methods associated with more traditional review processes [77-79]. Hence, a rapid review assesses what is already known in a given area within a relatively short period.

Our search strategy, detailed in Textbox 1, was developed in collaboration with a health sciences librarian and performed within the MEDLINE and Embase databases on February 5, 2022. Recognizing the dynamic nature of our study field, we conducted 2 subsequent updates to our search: the first on April 22, 2022, and the second on October 30, 2023. The strategy also included searches within reference lists and websites (eg, Google Scholar) for relevant material. We exported our search records to EndNote (Clarivate).

Our search was limited to records published in English, as suggested by the Cochrane rapid reviews guide [80], from 2017 to 2023. This time frame was chosen based on preliminary searches that indicated that the largest number of relevant articles was published during this period [81]. Furthermore, it allowed us to focus on chatbots incorporating more recent technological advancements. No limitations were set based on the study population.

**Figure 1:**
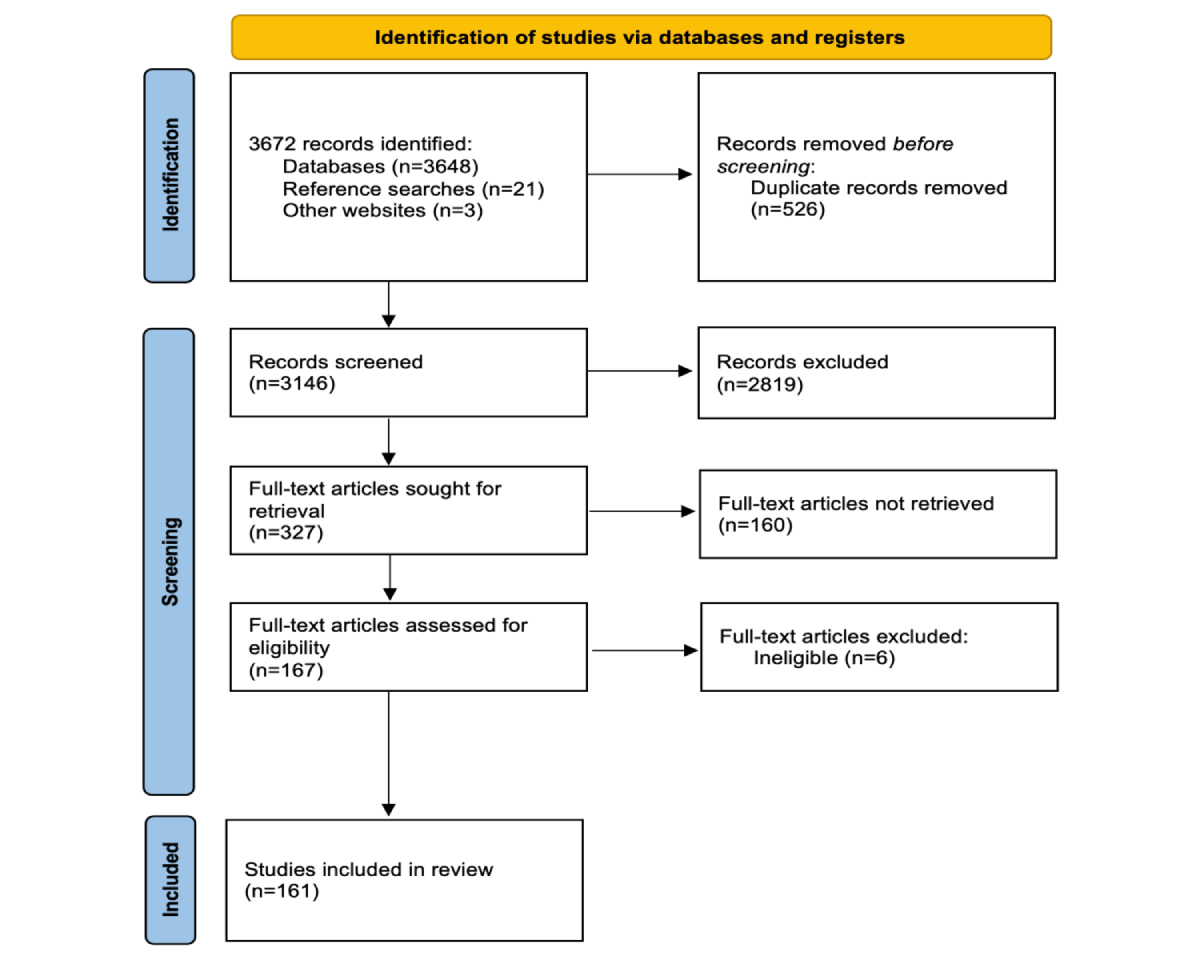
PRISMA (Preferred Reporting Items for Systematic Reviews and Meta-Analyses) flowchart showing the number of studies identified, screened, assessed for eligibility, and included in the final analysis.

Our rapid review adheres to the PRISMA (Preferred Reporting Items for Systematic Reviews and Meta-Analyses) guidelines, as depicted in Figure 1 [82].

**Search strategy for MEDLINE and Embase.**

**Search terms**

- user-computer interface/or (Chatbot* or chat bot* or User-Computer Interface* or (conversational adj2 (agent* or assistant*)).mp
- Limit 1 to yr = “2017 - Current”
- Limit 2 to English

### Study Selection

We included primary research studies that used text- or voice-based tailored chatbots as interventions within the health care system or as a means to deliver interventions. These studies report original data on the roles and benefits of chatbots in the health care setting.

Studies not meeting the inclusion criteria were excluded, as were studies reporting any of the following: engineering or computer science data, preintervention data about future initiatives such as protocols, and studies in the preintervention or predevelopment phase. We also excluded interventions based solely on nonbehavioral actions such as gestures and facial expressions without text or voice interaction, interactions with an actual robot (as opposed to a conversational interface), and virtual reality chatbots. In addition, abstracts lacking sufficient details were excluded.

### Data Extraction and Synthesis

Two reviewers (ML and YM) dual-screened 15% of the titles and abstracts and full texts to calculate the percentage agreement and interrater reliability, using Cohen κ [83]. Any discrepancies were resolved through discussion. ML conducted all remaining screenings. Data extraction was performed using Microsoft Office 365 (Excel and Word), capturing key study characteristics, including title, authors, month and year of publication, journal, study design, chatbot users, the chatbot’s medical specialty, whether the chatbot uses AI or is animated, and country of origin. In addition, we extracted information about the roles of chatbots, their benefits to health care, and their limitations. We categorized the source data into empirical and indicative data. This distinction reflects the 2-fold impact of contributions to the field: the actual findings demonstrate concrete evidence about the roles, users, benefits, and limitations of existing chatbots, while the authors’ discussion extends the conversation beyond current applications, providing perspective on the potential impacts, challenges, and future directions of health care chatbots, thus more comprehensively rounding out our assessment.

To synthesize these diverse pieces of information, relevant data underwent content analysis to generate subcategories, categories, and overarching themes [84].

While our research centers on chatbots, we have chosen to use the number of studies, rather than the chatbots themselves, as the basis for presenting most of our results. This approach accounts for the diverse adaptations to the identified chatbots across different contexts. Many of the chatbots we studied were modified to serve varied roles; cater to different user groups; and, in some cases, were given entirely different names in separate studies, as indicated in the *Results* section. Importantly, we noticed that a given study could contribute to multiple categories, indicating the flexible and interconnected characteristics of chatbot roles, users, benefits, or limitations. By focusing on the individual studies, we capture a more detailed and context-specific understanding of each chatbot’s functionality and versatility, which would be obscured if we merely counted each chatbot once, regardless of its various adaptations.

## Results

### Database Searches

Our search yielded 3672 records (databases: n=3146, 85.68%; reference searches: n=21, 0.57%; and other websites: n=3, 0.08%). After removing 526 (14.32%) duplicates from the 3672 records, 3122 (85.02%) records remained for title and abstract screening. During this screening phase, we achieved a 97% agreement rate and a Cohen κ value of 0.85, indicating substantial agreement beyond chance. Subsequently, of the 3146 records, 327 (10.39%) full texts were reviewed [85-245] (Figure 1), with 94% agreement and a Cohen κ value of 0.88 among the reviewers. Interrater reliability between the 2 reviewers, covering both the screening and final study inclusion as well as the data extraction process, ranged from 64% to 81%, indicating strong agreement [83]. This ensures the reliability and validity of the study selection and data extraction phases of our review.

After reviewing the 327 full texts, we ultimately included 161 (49.2%) studies that reported the roles and benefits of chatbots. All 161 studies reported on the roles of chatbots, 157 (97.5%) mentioned their benefits, and 157 (97.5%) addressed their limitations. Each study also reported on the user group or groups of focus that the chatbot was designed to assist.

### Origins of the Included Studies

**Figure 2:**
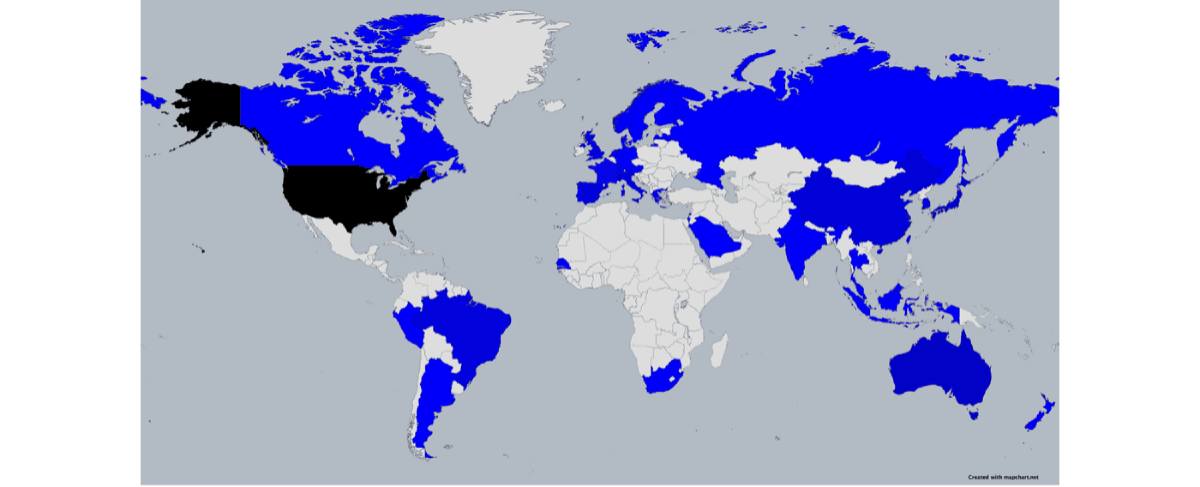
Map showing the countries that contributed the studies.

More than a quarter of the studies originated from the United States (46/161, 28.6%; Figure 2). China (15/161, 9.3%), Australia (10/161, 6.2%), Japan (9/161, 5.6%), and Spain (7/161, 4.3%) followed. Of the 161 studies, Italy, Switzerland, the United Kingdom, Singapore, Brazil, and South Korea each contributed 6 (3.7%), France and the Netherlands each contributed 4 (2.5%), while New Zealand, Greece, Russia, Norway, Malaysia, India, Senegal, Peru, Portugal, Canada, Latvia, South Africa, Indonesia, Argentina, Thailand, Saudi Arabia, Germany, and Austria each contributed 1 (0.6%) study. Notably, some studies were multinational; for instance, 1 (0.6%) of the 161 studies included Switzerland, Austria, and Germany; another included Northern Ireland, the Republic of Ireland, Scotland, Sweden, and Finland; yet another included Thailand, China, and Singapore; another study included India, North America, and the United Kingdom; a study included Finland, Denmark, and the Netherlands; another included Norway and Switzerland; and an additional study included the Netherlands and Scotland. Collectively, these 7 multinational studies account for 4.3% of the 161 included studies.

In our review of 161 studies, certain chatbots were the focus of multiple studies, particularly in the United States, Australia, South Korea, Switzerland, New Zealand, and Singapore; for instance, 2 specific chatbots were each the subject of 4 (2.5%) of the 161 studies (Gabby [94,99,101,114] and Woebot [86,92,119,173]). In addition, 11 chatbots were each studied twice (Todaki [90,102], GAMBOT [97,133], Laura [98,121], Vik [108,186], Termbot [151,195], ChatPal [158,168], a chatbot in a virtual ward [180,194], Corowa-kun [181,197], Dokbot [189,192], BotMaria [193,205], and COUCH [236,237]). Among these, a unique situation was observed in 5 (3.1%) of the 161 studies where the same original chatbot was presented under 5 different names [89,104,107,124,240]. These studies often shared several coauthors, indicating a common origin but with adaptations for different populations and roles. However, it is important to note that not all studies with mutual coauthors clearly indicated a shared origin of the chatbots.

### Chatbot Roles

#### Overview

All studies stated the role or roles of the chatbot used, with at least 1 role per study. Our analysis yielded 14 subcategories of primary roles (presented in italics), grouped into 5 categories, which were organized into 2 overarching themes, as summarized in Table 1.

**Table 1:** Health care chatbot roles (n=161).

Theme, category, and subcategory				Studies, n (%)
**Delivery of remote health services**				
	**Patient support and care management**			
		Mental health support	46 (28.6)	
		Counseling and treatment advice	26 (16.1)	
		Self-management and monitoring for chronic conditions	22 (13.7)	
		Triaging, screening, risk assessment, and referral	14 (8.7)	
		Self-care and monitoring for COVID-19 symptoms	8 (5)	
		Rehabilitation guidance	8 (5)	
		Reminders	7 (4.3)	
	**Education and skills building**			
		Health literacy	23 (14.3)	
		Medical education and clinical skills for health care professionals and medical students	12 (7.5)	
		Psychoeducation	5 (3)	
	**Health behavior promotion**			
		Healthy lifestyle behavior	30 (18.6)	
		Self-monitoring for health behavior change	6 (3.7)	
**Provision of administrative assistance to** **health care** **providers**				
	**Health-related administrative tasks**			
		Data collection and storage in patient electronic medical records	6 (3.7)	
	**Research purposes**			
		Recruitment and data collection	3 (1.9)	

#### Theme 1: Delivery of Remote Health Services

This theme refers to health services offered at a distance as an alternative or complement to the usual on-site modes of care delivery. It includes 3 categories and 7 subcategories of roles, with 158 (98.1%) of the 161 studies contributing to this theme.

##### Patient Support and Care Management

This category refers to the facilitation of medical consultations or the delivery of advice or support by providing counseling or treatment advice, triaging patients’ complaints, and fostering self-management and monitoring.

Overall, 103 (65.2%) of the 158 studies contributed to this category. Of these 103 studies, 46 (44.7%) mentioned using chatbots for *mental health support*, 26 (25.2%) reported providing *counseling and treatment advice* through chatbots, while 22 (21.4%) included chatbot use for improving *self-management or monitoring for chronic conditions*. Furthermore, of the 103 studies, 14 (13.6%) described chatbot use for *triaging, screening, risk assessment, and referral*; 8 (7.8%) studies each reported chatbot use for *self-care and monitoring for COVID-19 symptoms* and *rehabilitation guidance*; whereas 7 (6.8%) studies used chatbots to provide *reminders*.

##### Education and Skills Building

This category included the dissemination of educational material or medical information or skills development material (eg, exercising and using a medical device) for users, including patients, health care providers, or nursing and medical students.

In all, 41 (25.9%) of the 158 studies contributed to this category. Of these 41 studies, 23 (56%) reported promoting *health literacy* of the targeted population with the chatbot, 12 (29%) reported using chatbots in *medical education and clinical skills for health care professionals and medical students*, and *psychoeducation* was reported by 5 (12%) studies to enhance mental well-being.

##### Health Behavior Promotion

This category included the promotion of healthy lifestyles such as physical activity, a healthy diet, or stress management. Of the 158 studies, 39 (24.7%) contributed to this category. Of these 39 studies, *healthy lifestyle behavior* was encouraged through the chatbot in 30 (77%), while 6 (15%) reported *self-monitoring for health behavior change* as a chatbot role.

#### Theme 2: Provision of Administrative Assistance to Health Care Providers

This theme refers to all types of administrative work carried out by the chatbots, grouped within 2 categories—health-related administrative tasks and research purposes—with 9 (5.6%) of the 161 studies contributing to this theme.

##### Health-Related Administrative Tasks

This category included the completion of health care providers’ routine administrative work, such as data collection (eg, medical history taking), data entry, or transferring data to patients’ medical records. Of the 9 studies, 6 (67%) reported using the chatbot for *data collection and storage in patient electronic medical records* and charts, as well as for patient-reported outcome data, which could be captured by chatbots to replace collection by health care providers.

##### Research Purposes

This category refers to chatbot use for the completion of research-related work such as participant recruitment, the consent process, or data collection through surveys. Of the 9 studies, 3 (33%) contributed to this category, reporting the use of chatbots for participants’ *recruitment and data collection* through a self-administered questionnaire, in addition to obtaining electronic consent from individuals to participate in the study.

### Chatbot Users

#### Overview

All 161 studies specified the intended chatbot user population. The content analysis yielded 21 subcategories of chatbot users (presented in italics), grouped into 8 broader categories of users, as summarized in Table 2.

**Table 2:** Intended health care chatbot users (n=161).

Category and subcategory			Studies, n (%)
**Health condition–focused groups**			
	Individual seekers of mental health support	23 (14.3)	
	Patients with chronic conditions	10 (6.2)	
	Patients with cancer	7 (4.3)	
	Recovering patients	6 (3.7)	
**Lifestyle and general well-being enthusiasts**			
	Healthy adults	44 (27.3)	
	General public	16 (9.9)	
	Lifestyle-improvement seekers	9 (5.6)	
**Demographic and family-centric groups**			
	Women	14 (8.7)	
	Parents and children	7 (4.3)	
	Families	4 (2.5)	
**Age-based user groups**			
	Older adults	11 (6.8)	
	Young seekers of mental health support	8 (5)	
	Children	4 (2.5)	
**Underserved populations**			
	Culturally diverse groups	14 (8.7)	
	Individuals with disabilities	8 (5)	
**Health care** **professionals and students**			
	Medical and nursing students	8 (5)	
	Health care professionals	7 (4.3)	
**Health-related–behavior-change seekers**			
	Behavioral change seekers	8 (5)	
	Individuals in addiction recovery	7 (4.3)	
**Educational and skills enhancement seekers**			
	Nonmedical professionals	8 (5)	
	Health care training users	7 (4.3)	

#### Lifestyle and General Well-Being Enthusiasts

This category, with 69 (42.9%) of the 161 studies, addressed individuals aiming to improve or maintain their health and well-being. Of these 69 studies, 44 (64%) focused on *healthy adults* (adults who are in good health, without any significant or chronic medical conditions). *General public* (16/69, 23%) targeted the broader and more inclusive population that encompasses all segments of the population, regardless of their health status. *Lifestyle-improvement seekers*, encompassing 9 (13%) of the 69 studies, included individuals motivated to change their lifestyle.

#### Health Condition–Focused Groups

This category, comprising 46 (28.6%) of the 161 studies, included patients with specific health conditions across 4 subcategories. Of these 46 studies, individuals seeking mental health support, the largest subcategory with 23 (50%) studies, referred to adults with conditions such as attention-deficit and panic symptoms. *Patients with chronic conditions* (10/46, 22%) focused on individuals with conditions such as irritable bowel syndrome and hypertension. *Patients with cancer* (7/46, 15%) targeted those with breast cancer and those at risk for hereditary cancer. *Recovering patients* (6/46, 13%) focused on patients in various stages of recovery.

#### Demographic and Family-Centric Groups

Addressing specific demographic groups and family dynamics, this category comprised 15.5% (25/161) of the included studies. *Women* (14/25, 56%) focused on women’s health issues. *Parents and children* (7/25, 28%) centered on the health issues of children and adolescents. *Families* (4/25, 16%) looked at family dynamics and health.

Unlike age-based groups that are defined solely by the age of individuals, demographic and family-centric groups consider a wider range of factors, including gender, family roles, and the interplay of relationships within a family unit.

#### Age-Based User Groups

With 23 (14.3%) of the 161 studies, this category targeted specific age groups or life stages. *Older adults* (11/23, 48%) focused on older adults and age-related health concerns. Young seekers of mental health support (8/23, 35%) focused on mental health support for young adults. *Children* (4/23, 17%) targeted health issues specific to children.

#### Underserved Populations

With 22 (13.7%) of the 161 studies, this category focused on inclusive and accessible health care. *Culturally diverse groups* (14/22, 64%) targeted ethnic and cultural groups. *Individuals with disabilities* (8/22, 36%) focused on the unique health care needs of people with disabilities.

#### Health Care Professionals and Students

Encompassing 15 (9.3%) of the 161 studies, this category targeted health care professionals and students. *Medical and nursing students* (8/15, 53%) covered educational aspects for students in medical and nursing fields. Health care professionals (7/15, 47%) focused on training and professional development with this group.

#### Health-Related–Behavior-Change Seekers

Comprising 15 (9.3%) of the 161 studies, this category focused on behavioral health and lifestyle changes. *Behavioral change seekers* (8/15, 53%) included studies on individuals seeking to change health-related behaviors. *Individuals in addiction recovery* (7/15, 47%) targeted those dealing with addictions.

#### Educational and Skills Enhancement Seekers

Comprising 15 (9.3%) of the 161 studies, this category involved the use of chatbots for educational purposes. *Nonmedical professionals* (8/15, 53%) focused on skills enhancement for various professionals. *Health care training users* (7/15, 47%) were concerned about chatbots being used to train health care professionals.

While the *health care professionals* subcategory within the *health care professionals and students* category focuses on the professional development and training of individuals in the health care field, the *educational and skills enhancement seekers* category addresses a broader spectrum of users, including nonmedical professionals, and emphasizes the role of chatbots as a tool for educational purposes across various sectors.

### Health Care Chatbot Benefits

#### Overview

Most of the studies (157/161, 97.5%) described the benefits of using chatbots in health care. The content analysis yielded 7 different subcategories of benefits (presented in italics), grouped into 5 categories, which were organized into 2 broad themes, as summarized in Table 3.

**Table 3:** Reported health care chatbot benefits (n=157).

Theme, category, and subcategory				Studies, n (%)
**Improvement of** **health care** **quality**				
	**Improvement in health outcomes and patient management**			
		Improved mental health and well-being	42 (26.8)	
		Enhanced self-management	15 (9.6)	
		Improved physical health	8 (5.1)	
	**Promotion of patient-centered care and health equity**			
		Increased accessibility and reach of health care	60 (38.2)	
		Engaged and satisfied users	16 (10.2)	
		Supported groups considered vulnerable and reduced biases in health care delivery	4 (2.5)	
**Efficiency and cost-effectiveness in** **health care** **delivery**				
	**Optimization of resources**			
		Saved time and cost of health interventions	75 (47.8)	
	Scalability of health interventions			4 (2.5)
	Data quality and research support			4 (2.5)

#### Theme 1: Improvement of Health Care Quality

This theme refers to the processes of enhancing the standards, personalization, and accessibility of health care services delivered to the targeted chatbot users. It included 6 subcategories grouped into 2 categories of benefits, with 121 (77.1%) of the 157 studies contributing to the overarching theme.

##### Improvement in Health Outcomes and Patient Management

Of the 121 studies in this category, 65 (53.7%) addressed the benefits of chatbots to improve health outcomes and patient management. Of these 65 studies, 42 (65%) reported on *improved mental health and well-being*, 15 (23%) reported on *enhanced self-management*, and 8 (12.3%) reported on *improved physical health* as outcomes of using chatbots.

##### Personalization Through Patient-Centered and Equitable Care

Of the 121 studies, 62 (51.2%) reported on promoting personalization through patient-centered and equitable care. Chatbot personalization refers to customizing its interactions, content, and functionalities to suit individual needs and preferences, ensuring that it provides relevant, user-specific advice and support, enhancing its effectiveness and user experience. Health equity refers to minimizing disparities and inequality based on the social determinants of health, including differences between groups in terms of socioeconomic factors, gender, and ethnicity [246]. Patient-centered care addresses patients’ specific health care needs and concerns, improving the quality of personal, professional, and organizational relationships and aiding patients to actively participate in their own care [247,248].

Of the 62 studies, 60 (97%) discussed chatbot use benefits in terms of *increased accessibility and reach of health care* by helping engage diverse populations to access health services for minor health concerns that do not require emergency visits, with convenience and 24/7 availability.

Moreover, 16 (26%) of the 62 studies discussed using a chatbot to achieve *engaged and satisfied users*. In these studies, user acceptance was assessed by measuring the users’ positive feedback and their willingness to use the chatbot. This was often gauged through surveys or user feedback sessions after the interaction. The studies also highlighted that friendly interactions facilitated by the chatbot could enhance self-disclosure, further contributing to user satisfaction and engagement.

Of the 62 studies, 4 (6%) described chatbot use benefits for *supported groups considered vulnerable and reduced biases in health care delivery*, particularly for groups considered marginalized (eg, Black women and older users) facing stigma in health care settings and for people with low technological literacy.

#### Theme 2: Efficiency and Cost-Effectiveness in Health Care Delivery

##### Overview

This theme refers to chatbot use as favoring efficient care for targeted users. Providing efficient care means producing desired results with minimal or no waste of time, costs, materials, or personnel [249]. Three categories of benefits contributed to this overarching theme.

##### Optimization of Resources

In all, 75 (47.8%) of the 157 studies indicated reduced administrative or financial burdens for the health care system through chatbots because they can help relieve the burden of managing chronic health conditions, staffing shortage, and overwhelmed primary care settings. These studies indicated that chatbots could provide *saved time and cost of health interventions*, especially compared to other routine interventions.

##### Scalability of Health Interventions

Of the 157 studies, 4 (2.5%) indicated the feasibility of using chatbots for the implementation of large-scale health interventions to capture and assess large-scale public health situations, providing evidence for researchers and policy makers. The studies also addressed the significance of user data collected during the COVID-19 pandemic to evaluate the public health situation and aid decision-making by policy makers, public health authorities, and researchers.

##### Data Quality and Research Support

Of the 157 studies, 4 (2.5%) pointed out the benefits of enhancing data collection and clinical research quality by chatbots, providing timely, consistent, and standardized data collection, reducing human error, increasing patient engagement, and assisting in recruiting a diverse participant pool.

### Health Care Chatbot Limitations

#### Overview

Most of the studies (157/161, 97.5%) identified specific limitations of chatbots in health care, presented as 12 subcategories grouped into 5 categories, as summarized in Table 4.

**Table 4:** Reported health care chatbot limitations (n=157).

Category and subcategory		Studies, n (%)
**Challenges in user experience and overreliance**		
	Overconfidence and overreliance	154 (98.1)
	Usability and accessibility issues	135 (86)
**Technical challenges**		
	Complexity of effective language and communication processing	24 (15.3)
	Limitations in empathy and personal connection	17 (10.8)
	Challenges with resource allocation and cost efficiency	2 (1.3)
**Medicolegal and safety concerns**		
	Regulatory and legal issues	3 (1.9)
	Concerns about content and information quality	2 (1.3)
	Challenges in emergency response and expertise	2 (1.3)
**Societal and economic challenges**		
	Social, economic, and political challenges	5 (3.2)
	Issues of inequality in accessibility	4 (2.5)
**Ethical challenges**		
	Privacy and confidentiality concerns	2 (1.3)
	Ethical and safety concerns	2 (1.3)

#### Challenges in User Experience and Overreliance

A total of 157 (97.5%) of the 161 studies contributed to this category, addressing the tendency of *overconfidence and overreliance* among users who overestimate the capabilities of chatbots or rely excessively on them for health care needs, as noted in 154 (98.1%) studies. Overconfidence in chatbots can lead to users substituting professional medical advice with chatbot suggestions, while overreliance might result in users neglecting other essential aspects of health care or disregarding the need for human health care professional intervention. This subcategory highlights the importance of maintaining a balanced perspective on the capabilities and limitations of chatbots in health care contexts.

In addition, this category encompasses the *usability and accessibility issues* related to the ease with which users can interact with chatbots and the extent to which these chatbots are accessible to a diverse range of users, as referred to in most of the studies (135/157, 86%). It includes considerations of user interface design, the intuitiveness of chatbot interactions, the chatbots’ adaptability to different user needs, and their accessibility to individuals with varying levels of technology savviness or disabilities. Challenges in this category can lead to user dissatisfaction, reduced effectiveness of the chatbot, and potentially lower engagement with the health care service it provides.

#### Technical Challenges

This category refers to the broad spectrum of technological difficulties encountered in the design, development, and implementation of these systems, with 32 (20.1%) of the 157 studies contributing to it. This category underscores the need for sophisticated technology that can handle the nuances of health care communication and patient interaction while being accessible and practical for real-world application.

It includes the *complexity of effective language and communication processing*, as noted in 24 (75%) of the 32 studies, to ensure accurate and relevant medical information, as well as the chatbot’s ability to understand and respond to a range of user inputs, including those related to emotional states and complex health care queries.

The limitations extend to *challenges in empathy and personal connection*, which refer to the difficulties chatbots face in simulating human conversations and establishing rapport with users. This is a critical aspect in health care settings where patient trust and comfort are paramount, as highlighted in 17 (53%) of the 32 studies.

In addition, this category involves considering the *challenges with resource allocation and cost efficiency* of developing and maintaining these systems to ensure that they are not only technologically advanced but also financially viable and sustainable, as indicated in 2 (6%) of the 32 studies.

#### Medicolegal and Safety Concerns

With 6 (3.8%) of the 157 contributing studies, this category includes *regulatory and legal issues* encompassing the implications of chatbot advice and overall patient safety, as highlighted in 3 (50%) studies. These issues include chatbots’ compliance with health care regulations and patient privacy laws, liability for misdiagnosis or inadequate advice, and the need for specific regulatory guidelines for their development and application.

Furthermore, challenges extend to *concerns about content and information quality*, such as the medical accuracy of information provided by chatbots (eg, the potential for misdiagnosis) and the reliability of medical content. It also concerns limitations tied to the chatbot’s *challenges in emergency response and expertise* capabilities. Each of these subcategories was noted in 2 (33%) of the 6 studies.

#### Societal and Economic Challenges

This category refers to the wider implications of health care chatbots on the broader societal context and the economy, with 5 (3.2%) of the 157 contributing studies. It covers the influence of social, political, and economic factors on the adoption and effectiveness of chatbots in different communities.

It includes *social, economic, and political challenges* and considerations, as noted in all 5 studies. This subcategory scrutinizes the challenges arising from the integration of chatbots into the health care system, such as potential shifts in social norms, and the influence on economic policies and political decision-making in health care.

This category also includes *issues of inequality in accessibility*, as highlighted in 4 (80%) of the 5 studies. This subcategory delves into the challenges related to unequal access to chatbot technology. It focuses on how chatbots might inadvertently exacerbate existing disparities in health care, particularly for groups considered underprivileged, thereby highlighting the need for equitable distribution and accessibility of these technologies.

#### Ethical Challenges

This category deals with the ethical implications of using chatbots in health care, with 3 (1.9%) of the 157 studies contributing to it. It includes patient *privacy and confidentiality concerns* related to the use of patient data. This category also includes *ethical and safety concerns* encompassing the need to maintain transparency with users about the chatbot being a nonhuman agent and ensuring ethical standards in patient interactions. Each of these 2 subcategories was discussed in 2 (67%) of the 3 studies.

## Discussion

### Principal Findings

This rapid review revealed that chatbot roles in health care are diverse, ranging from patient support to administrative tasks, and they show great promise in improving health care accessibility, especially for groups considered marginalized. It also highlighted critical gaps in the literature, which are addressed in the following subsections.

#### Global Trends in Chatbot Research Indicate Its Predominance in Higher-Income Countries and Opportunities in Lower-Income Regions

With 35 countries represented by the studies in this review, the topic is clearly of global interest. However, more than a quarter of the included studies (46/161, 28.6%) originated from the United States, with the remainder conducted in high- or upper–middle-income countries across North America, Europe, and parts of Asia [250]. The concentration of chatbot research in high-income countries reflects underlying disparities with low- or lower–middle-income countries, particularly in parts of Africa, South America, and certain regions in Asia, in terms of technology access and health care investment. This gap highlights the need for more research focused on these regions, considering their unique digital infrastructure and resource challenges, to democratize health technology and address chronic conditions and health literacy [20,251-254].

#### Chatbots Have Varied Roles in the Enhancement of Health Care Delivery and User-Centric Services

Our review underscores the transformative roles of chatbots in health care, particularly in delivering remote health services and enhancing patient support, care management, and mental health support. Consistent with previous literature [254-257], our findings affirm chatbots’ potential to improve health care accessibility and patient management. The findings’ emphasis on education and skills building, particularly to enhance health literacy (which aligned with past literature [255,258]) and to support behavioral change (also highlighted by past research [255]), aligns with the growing need for patient empowerment in health care. The administrative efficiency of chatbots, noted in our review, resonates with previous findings [23,35,255,258] on the importance of resource optimization in health care settings.

Our findings indicate that chatbots also play a key role in facilitating clinical research, consistent with past work [259], a potential that needs further exploration, especially considering AI’s evolving role in health care [72,259-262].

#### The Diverse User Base of Chatbots Shows Their Potential to Support Equity and Bridge the Access Gap in Health Care Services

Our analysis indicates a broad and diverse user base for health care chatbots. From individuals focused on general well-being to those with specific health conditions, chatbots have been designed to cater to a wide array of needs. Notably, their use by demographic and family-centric groups and their accessibility to underserved populations underline the inclusive capacity of chatbots and their role in enhancing health care access and equity, especially for groups considered marginalized, in line with existing research [12,67,255,263-265].

In addition, our findings show the significant use of chatbots in mental health support for various age groups, reflecting the pressing need for accessible mental health services highlighted by others [4,8,12-17,29,30].

Furthermore, chatbots have emerged as tools for reducing stigma [12,265], linking users to health services [266-268], and protecting sensitive information [269]. Their empathetic and multilingual capabilities, as seen in our results [107,111,112,120,122,126-128,132] and past literature [270-276], are vital to reach diverse populations. They are particularly critical in light of the digital divide and the need for inclusive and accessible health care solutions [254,258,263,277,278].

#### The Use of AI in Chatbots Is a Promising but Still Evolving Field

The studies included in our review show a substantial number of AI-based chatbots, with fewer relying on non-AI platforms. AI in health care is recognized for its potential to improve health outcomes and the quality of life globally [260]. Given advances in machine learning and AI, expanding the scope of chatbots is expected to cause a mutation in their role in the health care system to assist clinicians and potentially take over some of their duties [72,261,262]. The synergy between big data and AI, coupled with the increasing availability of data in health care, suggests that AI-based chatbots could effectively use extensive health care data [259,279]. This aligns with 1 (0.6%) of the 161 included studies [94], which discusses the use of collected data as a key benefit of chatbots. However, ethical considerations such as data privacy and algorithmic biases must be addressed for responsible AI deployment, crucial for maintaining trust and fairness [73].

Studies included in this review indicate that using avatars in these chatbots to simulate social behaviors can enhance user engagement and trust. This form of chatbot technology is particularly appealing in patient interactions and medical education to establish trust and therapeutic alliances between health care professionals and patients and to improve the communication skills of medical students and health care professionals [118,123,130,131,280].

Balancing AI’s benefits to enhance data use and user interactions with its ethical concerns, including data privacy and algorithmic bias, is crucial for its implementation, shaping the future of patient care and medical education in an innovative and ethically sound way.

#### Despite the Potential Revolutionary Roles of Chatbots in Health Care, Critical Challenges and Limitations Exist

This review stresses that despite chatbots’ roles and benefits, their use comes with various challenges, including ethical, technical, medicolegal, and user experience concerns, as also discussed in past literature [3-5,23,25,30,72,74,95,281,282].

While the studies included in our review have highlighted chatbot use to address minor health concerns and provide off-hour information, there is a noticeable gap in evaluating their technical limitations, especially in complex health care scenarios, as underscored by past literature [3-5,23,25,30,72,74,95,281,282]. This raises concerns about patient safety and the accuracy of health management, emphasizing the need for comprehensive assessment and iterative improvement of chatbot technologies [22,25,68,72,95,254,283].

The findings in our review indicate the regulatory and ethical landscape for chatbots as another area of concern. This agrees with past studies highlighting the need for ethical use, data privacy, and transparent communication about chatbots’ capabilities and limitations [4,73,74,254,281,284,285]. The absence of specific laws and regulations addressing health care chatbot use introduces risks around liability and medicolegal issues [72,286,287]. These challenges are further complicated by ethical dilemmas, such as privacy and confidentiality in nonanonymous interactions [71,72,288,289] and safety concerns in medical emergencies due to limited chatbot expertise [72].

Technical issues identified by this review, including difficulty in language processing and a lack of empathic response, can lead to trust issues and increased clinical workload and align with past literature [3-5,68,72,73,280,290]. Overreliance on chatbots for self-diagnosis and health care decisions may lead to misjudgments, potentially exacerbating health issues [4,68,73]. In addition, the financial motives of private companies in the health sector raise ethical concerns about the primary purpose and application of health chatbots [73]. The requirement for sophisticated AI technology also implies increased demands on human resource expertise and storage services, potentially escalating costs [73,287].

Our results indicate that chatbots serve a wide range of populations from various groups in terms of age, gender, ethnicity, and socioeconomic and educational status due to their promising acceptability and usability [291]. However, the digital divide [292-294], algorithmic ethical concerns [295], and the potential misuse of chatbots in replacing established health services [296] present risks. These factors, along with social, economic, and political influences [297], could inadvertently widen health disparities, highlighting the importance of inclusive and equitable chatbot development and deployment.

The discussion on health care chatbots is fundamentally about their potential and promise, grounded in our exploration of current studies and developments. These digital tools could significantly enhance health care access, service quality, and efficiency. However, realizing their full potential hinges on addressing challenges such as ethical AI use, data privacy, and integration with health care systems.

Efforts moving forward should concentrate on incorporating AI responsibly and designing chatbots that cater to all user demographics, ensuring equitable health care access. Collaboration across technology, health care, and policy sectors is crucial to establish ethical guidelines and confirm chatbots’ efficacy and safety. Successfully navigating these challenges will enable chatbots to fulfill their promising role in health care, contributing to a more accessible and patient-focused system.

### Limitations

This review, while insightful, is not without its limitations. Although rapid and systematic reviews are often considered comparable in their conclusions, each methodology has its own set of constraints [289,298]. Specifically, this rapid review was limited by a noncomprehensive search strategy that included only 2 databases. In addition, the inclusion criteria were restricted by date and language, which potentially led to the exclusion of some pertinent studies. Another limitation was the concentration of screening and analysis tasks on a single reviewer (ML), which might have introduced bias or overlooked nuances in the data. Moreover, a formal quality appraisal of the included studies was not conducted due to the descriptive nature of this review. Consequently, this limitation may affect the depth of understanding and the strength of the conclusions drawn.

One critical aspect of our methodology was the combination of empirical findings and opinion-based data from the discussions in the included studies. We did not distinguish between these 2 types of data but rather treated them as a unified source of information. This approach, while allowing for a comprehensive overview of chatbots in health care, might have led to a potential bias in favor of chatbot benefits because both empirical results and positive speculative insights were reported together. However, this potential bias is somewhat mitigated by our consistent reporting of the challenges associated with chatbots, as identified in the included studies. By presenting both the potential benefits and the challenges, we aimed to offer a balanced view, reducing the likelihood of a 1-sided interpretation favoring chatbot benefits.

In addition, this review might have overestimated the results due to the dependence on the discussion sections of each study, which may have overcounted the results and miscounted those that may have disagreed or contradicted the results of these included studies. However, this did not significantly impact the study’s aim to provide an exploratory and descriptive overview of health care chatbots, mapping out the landscape of their applications in health care. In such a context, a broad, inclusive approach that captures diverse opinions and trends is more important than precise quantification.

Moreover, one of the potential limitations of this review is the exclusion of generative AI and LLMs such as ChatGPT. However, among the studies we reviewed, a standout comparison involved a health care chatbot, specialized in medical terminology, and ChatGPT. This unique comparison serves to highlight the advanced capabilities of LLMs such as ChatGPT in enhancing the delivery and accuracy of remote health services [59,75]. Nonetheless, a significant challenge persists in guaranteeing the contextual relevance and appropriateness of chatbot responses, particularly in intricate medical scenarios [59,60]. In addition, the personalization of health care interactions and the precision of information provided by these AI-driven systems are critical areas necessitating extensive future research and rigorous evaluation of their outputs [59,60,299].

Finally, the results were presented solely as a narrative summary [77], which might limit the breadth of perspectives and interpretations that a more diverse methodological approach could have provided. Nevertheless, the inclusion of both benefits and challenges in our reporting suggests that the review may not be significantly biased toward a positive portrayal of chatbots, providing a more nuanced understanding of their role in health care.

### Conclusions

This review underscores the significant potential of chatbots in health care, evident in their diverse roles, benefits, and user populations. In addition, it explores the current limitations and challenges of chatbot development and implementation in health care. Finally, it underscores significant research gaps in the field. As such, this review aims to contribute to academic discourse on this important topic and offer insights into the effective design, implementation, and investigation of chatbots in health care.

## Abbreviations

AI: artificial intelligence
LLM: large language model
PRISMA: Preferred Reporting Items for Systematic Reviews and Meta-Analyses
